# Fasting inhibits excitatory synaptic input on paraventricular oxytocin neurons *via* neuropeptide Y and Y1 receptor, inducing rebound hyperphagia, and weight gain

**DOI:** 10.3389/fnut.2022.994827

**Published:** 2022-10-19

**Authors:** Lei Wang, Shigetomo Suyama, Samantha A. Lee, Yoichi Ueta, Yutaka Seino, Geoffrey W. G. Sharp, Toshihiko Yada

**Affiliations:** ^1^Division of Integrative Physiology, Department of Physiology, Jichi Medical University School of Medicine, Shimotsuke, Japan; ^2^Division of Integrative Physiology, Center for Integrative Physiology, Kansai Electric Power Medical Research Institute, Kyoto, Japan; ^3^Division of Diabetes, Metabolism and Endocrinology, Kobe University Graduate School of Medicine, Kobe, Japan; ^4^Department of Diabetes, Endocrinology and Metabolism/Rheumatology and Clinical Immunology, Gifu University Graduate School of Medicine, Gifu, Japan; ^5^Department of Physiology, School of Medicine, Keio University, Tokyo, Japan; ^6^Department of Biological Sciences, University of Manitoba, Winnipeg, MB, Canada; ^7^Department of Physiology, School of Medicine, University of Occupational and Environmental Health, Kitakyushu, Japan; ^8^Yutaka Seino Distinguished Center for Diabetes Research, Kansai Electric Power Medical Research Institute, Osaka, Japan; ^9^Department of Molecular Medicine, College of Veterinary Medicine, Cornell University, Ithaca, NY, United States

**Keywords:** food restriction, neuropeptide Y, Y1 receptor, paraventricular nucleus, oxytocin, synaptic plasticity, hysteresis, rebound

## Abstract

Fasting with varying intensities is used to treat obesity-related diseases. Re-feeding after fasting exhibits hyperphagia and often rebound weight gain. However, the mechanisms underlying the hyperphagia and rebound remain elusive. Here we show that 24 h food restriction (24 h FR) and milder 50% FR, both depress synaptic transmission in the hypothalamic paraventricular nucleus (PVN) and induce acute hyperphagia in rats. 24 h FR is followed by weight rebound but 50% FR is not. Orexigenic neuropeptide Y (NPY) *via* the Y1 receptor (Y1R) inhibited the miniature excitatory postsynaptic current (mEPSC) on anorexigenic oxytocin neurons in the PVN. 24 h FR and 50% FR activated this neuronal pathway to induce acute hyperphagia on Days 1–3 and Days 1–2 after FR, respectively. 24 h FR induced large mEPSC depression, recurrent hyperphagia on Days 9–12 and rebound weight gain on Days 12–17, whereas 50% FR induced moderate mEPSC depression and sustained weight reduction. Transverse data analysis on Day 1 after 24 h FR and 50% FR demonstrated saturation kinetics for the mEPSC depression-hyperphagiacurve, implying hysteresis. The results reveal FR-driven synaptic plasticity in the NPY-Y1R-oxytocin neurocircuit that drives acute hyperphagia. FR with the intensity that regulates the synapse-feeding relay without hysteresis is the key for successful dieting.

## Introduction

Fasting is experienced by humans because of environmental, social, religious, and medical reasons (1–3). Re-feeding after fasting exhibits acute, and occasionally chronic, hyperphagia (1–3). In the hypothalamic feeding centers, the projection of the arcuate nucleus (ARC) neurons expressing NPY and agouti-related peptide (AgRP) to the PVN, including the anorexigenic oxytocin (OXT) neurons (4, 5), plays a key role in stimulating feeding (6–9). Fasting acutely activates the ARC NPY/AgRP neurons (10, 11) and increases NPY content in PVN (12). This NPY pathway from the ARC to PVN serves to acutely stimulate feeding and restore body weight after fasting (6–9). However, its involvement in the long-term impact of fasting on feeding behavior and weight is unknown. These acute and long-term influences may involve an alteration of synapses (13–16). The precise role of synaptic plasticity in the fasting-induced alteration of feeding behavior and weight remains to be clarified. This study, using the OXT neuron-specific monomeric red fluorescent protein 1 transgenic (OXT mRFP) rats (17), explored whether NPY and Y receptor regulate synaptic currents on OXT neurons and, if so, whether this neuronal pathway is implicated in the effects of fasting. This study also explored how the different regimens of fasting, a strong regimen of 24 h food restriction (24 h FR) and milder 50% food restriction (50% FR), differentially influence synapses, feeding, and weight, in order to gain insights into the mechanisms for weight reduction vs. rebound after dieting.

## Materials and methods

### Animals

Male Wistar rats (Nihon SLC, Hamamatsu, Japan) aged 7–9 weeks, OXT-mRFP transgenic rats aged 6–7 weeks, and AVP eGFP (enhanced green fluorescent protein) transgenic rats (17) aged 7 weeks were used. All animals were housed individually or in groups of two to four rats in a temperature-controlled environment at 22–24 C under a 12 h light/dark cycle (19:30 lights off). All rats were fed conventional food (CE-2; Clea, Osaka, Japan) and provided with water *ad libitum*. Experimental procedures and care of animals were carried out according to the Jichi Medical University Animal Care and Use Committee and the Committee on Animal Experimentation of Kobe University.

### Preparation and treatment of hypothalamic slices

Rats were anesthetized with isoflurane and decapitated at around 10:00. The brains were rapidly removed and immersed in an ice-cold, carboxygenated (95% O_2_ and 5% CO_2_) high-mannitol solution containing (in mM): 229 mannitol, 3 KCl, 1 NaH_2_PO_4_, 26 NaHCO_3_, 0.5 CaCl_2_, 6 MgCl_2_, and 10 glucose, pH 7.3 with KOH. A block of tissue containing the hypothalamus was isolated and coronal slices (300 μm) were cut on a Vibratome. Slices were maintained in a holding chamber with carboxygenated artificial cerebrospinal fluid (aCSF) containing (in mM) 124 NaCl, 3 KCl, 1 MgCl_2_, 2 CaCl_2_, 1 NaH_2_PO_4_, 26 NaHCO_3_, and 2.5 glucose for about 40 min for recovery.

The slices were incubated *ex vivo* for 3 h with the following chemicals; NPY (10^–8^ M), Y1R antagonist GR231118 (0.5 μM), Y5R antagonist RA972 (0.1 μM), and/or an NMDA receptor antagonist, d-(-)-2-amino-5-phosphonopentanoic acid (AP5) (50 μM). After washing the slices, they were subjected to patch clamp recordings.

### Patch-clamp recording

Hypothalamic slices were moved to a recording chamber mounted on a BX51WI upright microscope equipped with video-enhanced infrared-differential interference contrast and were constantly perfused with aCSF (32^°^C) at a rate of 1.5–2 ml/min. Neurons were visualized with an Olympus Optical 40 × water-immersion lens. Whole-cell patch-clamp recording was performed under voltage clamp conditions as reported previously (15). Recordings from slices dissected at around 10:00 yielded consistent results during recording periods of several hours, indicating that the neuronal properties of the rats *in vivo* at the time of dissection were well retained in slices *ex vivo*, consistent with previous reports (13–16).

Oxytocin neurons in slices were identified by RFP fluorescence. The micropipettes were prepared using borosilicate glass (G-1.5 glass capillaries, Narishige) and a P-1000, Sutter Instrument Co micropipette puller. After being filled with pipette solution they had resistances of 3–9 MΩ. The composition of the pipette solution was as follows (in mM): 135 Cs-methanesulfonate, 2 MgCl_2_, 10 HEPES, 1.1 EGTA, 2 Mg-ATP, 0.3 Na_2_-GTP, 0.1 spermine, and 10 Na_2_-phosphocreatine, pH 7.3 with KOH. For mEPSC recording, 100 μM picrotoxin and 0.5 μM tetrodotoxin (TTX) were added in aCSF to inhibit GABAergic inputs and presynaptic action potentials, respectively. For AMPAR/NMDAR of evoked EPSC (eEPSC) recording, 5 mM QX-314 and 10 mM tetraethylammonium were added in the inner pipette solution to block sodium and potassium channels, respectively. AMPAR mediated eEPSC (AMPAR-eEPSC) was recorded with holding potential of –60 mV and with 100 μM picrotoxin in aCSF to inhibit GABAergic input. NMDAR mediated eEPSC (NMDAR-eEPSC) was recorded with a holding potential of +40 mV and with 100 μM picrotoxin and 10 μM 6-cyano-7-nitroquinoxaline-2,3-dione (CNQX) to inhibit GABAergic input and AMPAR-eEPSC, respectively. Both input resistance and series resistance were monitored throughout the experiments and the former was partially compensated. Only those recordings with stable series resistance and input resistance were accepted. All data were sampled at 10 kHz, filtered at 1–3 kHz and analyzed with pClamp10 (Axon instruments, Sunnyvale, CA, United States). Average and SEM of mEPSC amplitude was calculated from median value of each recording.

### Intra-paraventricular nucleus injection of Y1 receptor antagonist

**FIGURE 1:**
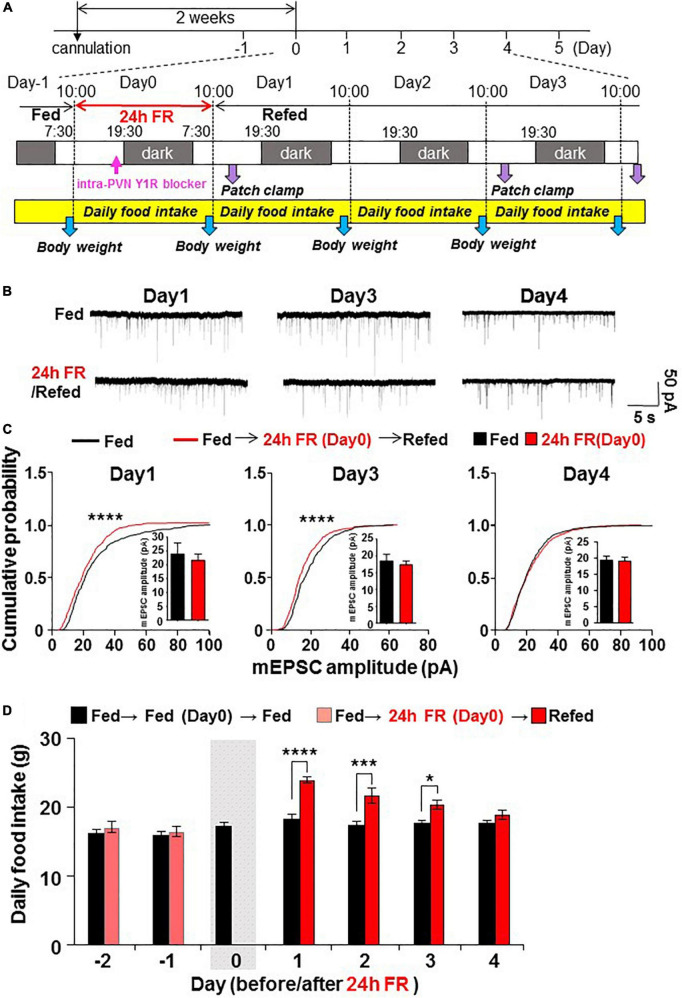
Twenty four hours food restriction (24 h FR) depresses synaptic currents on paraventricular nucleus (PVN) oxytocin (OXT) neurons and leads to hyperphagia on refeeding. **(A)** Protocol of the *in vivo* and *ex vivo* experiments. OXT mRFP rats were fasted for 24 h from 10:00 on Day 0 to 10:00 on Day 1. In Figures 5A–C, the Y1R antagonist was injected intra-PVN at 19:00 also on Day 0. Subsequently, hypothalamic slices were prepared and PVN OXT neurons were subjected to whole-cell patch-clamp recording on Days 1, 3, and 4 of re-feeding. Daily food intake (10:00–10:00 next day) and body weight were measured. **(B**,**C)** 24 h FR depressed mEPSC **(B)** and cumulative probability distribution of mEPSC amplitudes **(C)** in PVN OXT neurons on Days 1 and 3 but not Day 4 of re-feeding. ****P < 0.0001 by Kolmogorov–Smirnov test. *n* = 9 in each group. **(D)**, Daily food intake in OXT mRFP rats. 24 h FR induced daily hyperphagia on Days 1, 2, and 3 but not Day 4 of re-feeding. The gray shadow indicates the day of 24 h FR. ****P < 0.0001, ***P < 0.001, and *P < 0.05 by two-way ANOVA with *post-hoc* Sidak’s test. *n* = 5 in each group.

Male OXT mRFP rats aged 6 weeks were anesthetized by isoflurane inhalation solution (2–4%, Pfizer, Japan), placed on the stereotaxic frame (Model 900 Single Manipulator 100-micron, David Kopf Co., United States), and implanted with guide cannulae. The bilateral guide cannula (C235G-0.5/SPC/26G double cannula, Plastics One, United States) was inserted into the skull above the center of PVN (0.03 mm lateral to the bregma, 1.8 mm caudal and 7.4 mm below the surface of the skull). Rats were allowed to recover from surgery for at least 1 week with handling, before being subjected to tests. From 1 week after surgery, daily food intake was measured every day at 10:00 (Figure 1A). At 2 weeks after surgery, 0.3 μl saline (0.9%) or Y1R antagonist GR231118 (10 μg/5 μl, 0.3 μl) was intra-PVN injected at around 19:00 on Day 0.

### Protocols for 24 hour food restriction and 50% food restriction

Twenty four hour food restriction were performed from 10:00 on Day 0 to 10:00 on Day 1 (Figure 1A). In 50% FR experiments, daily food intake was measured from 3 days before Day 0 (Day-3) to Day 0, and averaged, and 50% of the average daily food intake over this period was given to the rats at 10:00 on Day 0.

### Measurements of food intake and body weight

In the studies of feeding behavior and patch-clamp recording, daily food intake was measured at 10:00 from 2 days before (Day-2) through 4 days after FR (Day 4). In studies for long-term measurements of daily food intake and weight, food intake was measured from Day-1 through Day 10 or 17.

### Statistical analysis

Data are presented as mean ± SEM. All groups showed normal variance and equal variances as a result of F or Bartlett’s tests. Statistical analysis was performed by unpaired *t*-test, ANOVA followed by *post-hoc* Tukey’s test, and two-way ANOVA followed by *post-hoc* Sidak’s test using Prism 7 (GraphPad Software, La Jolla, CA, United States), and Kolmogorov–Smirnov test for cumulative probability of EPSC amplitude using AxoGraphX (AxoGraph, Berkeley, CA, United States). *P* < 0.05 was considered statistically significant. More specific and detailed statistics are described in the corresponding figure legends.

## Results

### Twenty four hour food restriction depresses miniature excitatory postsynaptic current in paraventricular nucleus oxytocin neurons, induces hyperphagia, and rebound weight gain

Patch-clamp measurements were made of mEPSC on OXT neurons in PVN slices prepared from OXT-mRFP rats (15), combined with measurements of daily food intake. The rats were fasted for 24 h on day 0 and subsequently re-fed for 4 days (Figure 1A). 24 h FR, compared to fed controls, depressed mEPSC (Figure 1B) with significant (*P* < 0.0001, Kolmogorov–Smirnov test) reduction in the cumulative probability distribution of mEPSC amplitudes in PVN OXT neurons on Days 1 and 3 but not Day 4 of re-feeding (Figure 1C). 24 h FR tended to decrease the average mEPSCs amplitude on Day1 and Day3 but the changes were not statistically significant (Figure 1C). 24 h FR did not significantly alter the mEPSC frequency in PVN OXT neurons on Days 1, 3, and 4 (Supplementary Figure 2A). Daily food intake was significantly elevated on Days 1–3 (Fed vs. 24 h FR; Day 1, 18.15 ± 0.76 vs. 23.99 ± 0.39 g, *P* < 0.0001; Day 2, 17.36 ± 0.51 vs. 21.69 ± 1.07 g, *P* < 0.001; Day 3, 17.63 ± 0.53 vs. 20.37 ± 0.65 g, *P* < 0.05, two way-ANOVA followed by Sidak’s test, *n* = 5) but not Day 4 of re-feeding (Figure 1D). Thus, 24 h FR induced mEPSC inhibition in PVN OXT neurons and hyperphagia with the same time course. OXT and arginine vasopressin (AVP), expressed in the PVN and the supraoptic nucleus, share 78% amino acid homology and regulate several common functions including feeding (17, 18). In the PVN AVP neurons from the slices prepared from AVP eGFP rats, 24 h FR had no effect on mEPSC on Day 1 of re-feeding (data not shown), indicative of a selective effect of fasting on OXT neurons.

**FIGURE 2:**
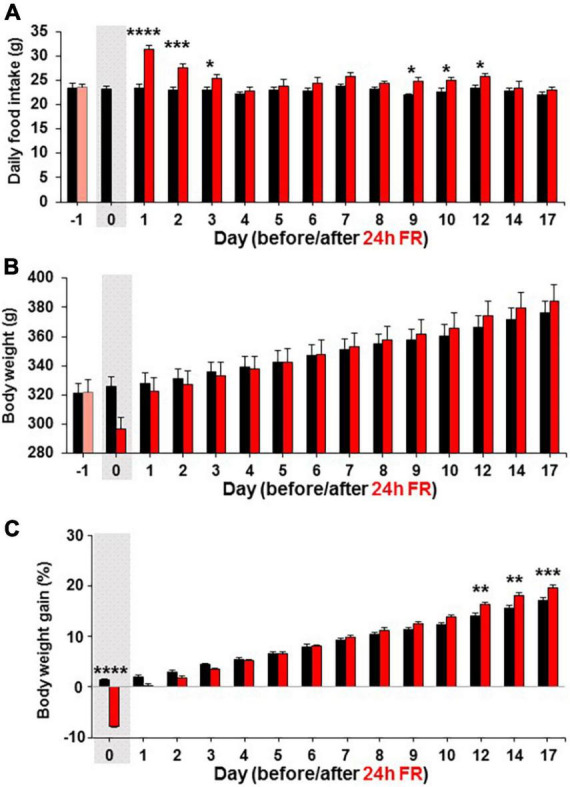
Twenty four hours food restriction (24 h FR) leads to hyperphagia on refeeding. Long-term effects of 24 h FR on daily food intake **(A)**, body weight **(B)**, and body weight gain **(C)** in Wistar rats. The body weight gain was determined by the change from the weight on Day-1. The gray shadows indicate the day of 24 h FR. *n* = 5–6. *P < 0.05, **P < 0.01, ***P < 0.001, and ****P < 0.0001 between 24 h FR vs. fed groups by two-way ANOVA followed by *post-hoc* Sidak’s test.

In separate experiments in Wistar rats, after 24 h FR, compared with fed control, daily food intake was significantly increased on Day 1–3 (Fed vs. 24 h FR; Day1, 23.46 ± 0.82 vs. 31.3 ± 0.85 g, *P* < 0.0001; Day 2, 22.98 ± 0.71 vs. 27.64 ± 0.75 g, *P* < 0.001; Day 3, 23.06 ± 0.43 vs. 25.39 ± 0.76 g, *P* < 0.05) and also Days 9–12 (Day 9, 21.97 ± 0.21 vs. 24.7 ± 0.89 g; Day 10, 22.68 ± 0.66 vs. 24.92 ± 0.65 g; Day 12, 23.37 ± 0.69 vs. 25.68 ± 0.72 g, *P* < 0.05, two way-ANOVA followed by Sidak’s test, *n* = 5–6; Figure 2A). Body weight was not different from control at any time up to Day 17 (Figure 2B). The body weight gain, determined by the change from the weight on Day-1, tended to be lower on days 1–4 catching up on day 5 compared to fed control as the rats were recovering from the fasting day weight loss, but was significantly higher on Days 12–17 (Fed vs. 24 h FR; Day 12, 15.60 ± 0.51 vs. 17.99 ± 0.58%, *P* < 0.01; Day 14, 16.08 ± 0.36 vs. 18.52 ± 0.45%, *P* < 0.01; Day 17, 17.13 ± 0.50 vs. 19.53 ± 0.62%, *P* < 0.001, two way-ANOVA followed by Sidak’s test, *n* = 5–6; Figure 2C). Thus, hyperphagia reappeared on Days 9–12 being accompanied by increases in the weight gain on Days 12–17, exhibiting the rebounds of feeding and weight.

These results showed that 24 h FR induced mEPSC inhibition selectively in PVN OXT neurons on Days 1–3, an action being paralleled with acute hyperphagia on Days 1–3 and followed by late hyperphagia and weight rebound. This observation prompted us to explore the mechanism of the fasting-induced mEPSC inhibition in PVN OXT neurons.

### Neuropeptide Y depresses miniature excitatory postsynaptic current in paraventricular nucleus oxytocin neurons *via N*-methyl-D-aspartic acid type glutamate receptor

**FIGURE 3:**
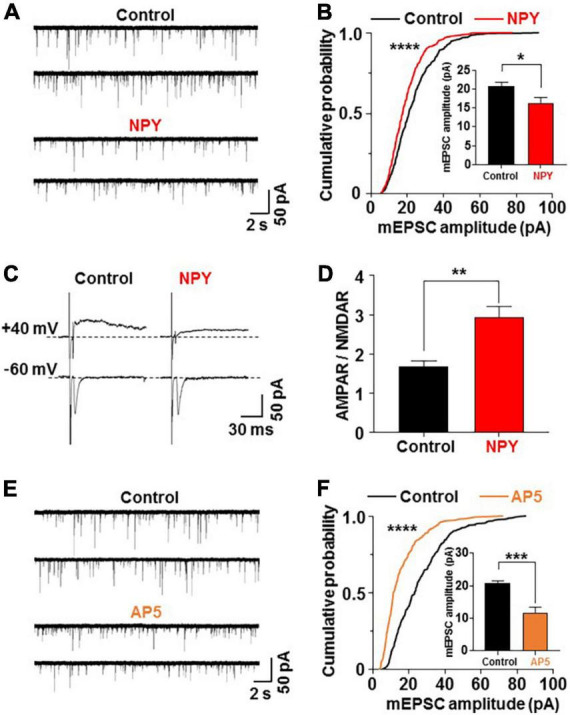
Neuropeptide Y (NPY) depresses miniature excitatory postsynaptic current (mEPSC) by inhibiting *N*-methyl-D-aspartic acid type glutamate receptor (NMDAR). Hypothalamic slices from oxytocin (OXT) mRFP rats were pretreated for 3 h without (control) and with NPY (10^– 8^ M) or the NMDAR antagonist AP5 (50 μM). Subsequently, paraventricular nucleus (PVN) OXT neurons were subjected to whole-cell patch-clamp recording. Upper and lower traces of mEPSC in each condition show results from two distinct OXT neurons. **(A,B)** Representative traces of mEPSC **(A)**, cumulative probability distribution of mEPSC amplitudes **(B)** and average amplitude of mEPSC (insert) **(B)** in PVN OXT neurons pretreated for 3 h without (control) or with 10^– 8^ M NPY. ****P < 0.0001 by Kolmogorov–Smirnov test. *P < 0.05 by unpaired *t*-test. *n* = 10 for control and *n* = 9 for NPY groups. **(C,D)** NMDAR-evoked EPSC (eEPSC) (upper traces) and AMPAR-eEPSC (lower traces) **(C)** and AMPAR-eEPSC/NMDAR-eEPSC ratio **(D)** in PVN OXT neurons pretreated for 3 h without (control) or with 10^– 8^ M NPY. **P < 0.01 by unpaired *t*-test. *n* = 7 for each group. **(E,F)** Representative traces of mEPSC **(E)**, cumulative probability distribution of mEPSC amplitudes **(B)** and average amplitude of mEPSC (insert) **(F)** in PVN OXT neurons preincubated without or with AP5. *n* = 6–7 for each group. ****P < 0.0001 by Kolmogorov–Smirnov test. ***P < 0.001 by unpaired *t*-test.

Negative energy balance is known to stimulate brain circuits that activate the orexigenic and energy conserving pathways. The most important neurons that regulate these processes are the NPY/AgRP neurons in the hypothalamic ARC (6). Among the hypothalamic orexigenic neuropeptides and their receptors that are regulated by fasting and re-feeding, the largest changes reportedly occur in genes associated with NPY and Y receptor systems (19). Hence, we first explored whether the NPY-Y receptor system drives the depression of mEPSC on PVN OXT neurons. Hypothalamic slices from OXT mRFP rats were preincubated without (control) or with 10^–8^ M NPY for 3 h, followed by whole-cell patch-clamp recording on PVN OXT neurons. NPY decreased the amplitude of mEPSC on OXT neurons (Figure 3A) by significantly (*P* < 0.0001, Kolmogorov–Smirnov test) left-shifting the distribution of the amplitude of mEPSC and decreasing the average amplitude (Control vs. NPY; 21.85 ± 0.92 vs. 15.83 ± 0.83 pA, *P* < 0.05, by unpaired *t*-test) (Figure 3B). NPY did not significantly alter the mEPSC frequency in PVN OXT neurons (Supplementary Figure 2B).

Our previous report (15) showed that the ratio of the α-amino-3-hydroxy-5-methyl-4-isoxazolepropionic acid type glutamate receptor (AMPAR)-mediated EPSC to the NMDAR-mediated EPSC in the PVN-OXT neuron was increased by 24 h FR. To examine the involvement of AMPAR and NMDAR in NPY inhibition of mEPSC on OXT neurons, we measured evoked EPSC (eEPSC) at -60 mV holding potential that reflects AMPAR-eEPSC, and eEPSC at +40 mV holding potential in the presence of the AMPAR blocker CNQX (10 μM) that reflects NMDAR-eEPSC (15). After treatment with NPY, the ratio of AMPAR-eEPSC/NMDAR-eEPSC (AMPAR/NMDAR) was significantly increased (Control vs. NPY; 1.71 ± 0.24 vs. 2.94 ± 0.33, *P* < 0.01, by unpaired *t*-test, *n* = 7; Figures 3C,D). On the other hand, mEPSC, which is primarily mediated by AMPAR, was reduced. Taken together, the increase in AMPAR/NMDAR suggested substantially greater reduction of NMDAR. These data support that NPY primarily inhibits NMDAR to suppress AMPAR. To confirm this, PVN slices were preincubated with an NMDA receptor antagonist AP5 (50 μM) for 3 h. The treatment with AP5 significantly reduced the amplitude of mEPSC on PVN OXT neurons (Figure 3E), left-shifted the distribution and reduced the average of mEPSC amplitude (Control vs. AP5; 20.75 ± 0.75 vs. 11.75 ± 1.69 pA, *P* < 0.001, by unpaired *t*-test, *n* = 6–7; Figure 3F), mimicking the effects of NPY. AP5 did not significantly alter the mEPSC frequency (Supplementary Figure 2B). These results show that NPY regulates EPSC on anorexigenic OXT neurons *via* mechanisms involving NMDAR regulation, which may trigger subsequent suppression of AMPAR mediated transmission. The NMDAR-mediated activation of hypothalamic orexigenic neurons was previously reported (13).

### Neuropeptide Y *via* Y1 receptor depresses miniature excitatory postsynaptic current in paraventricular nucleus oxytocin neurons

**FIGURE 4:**
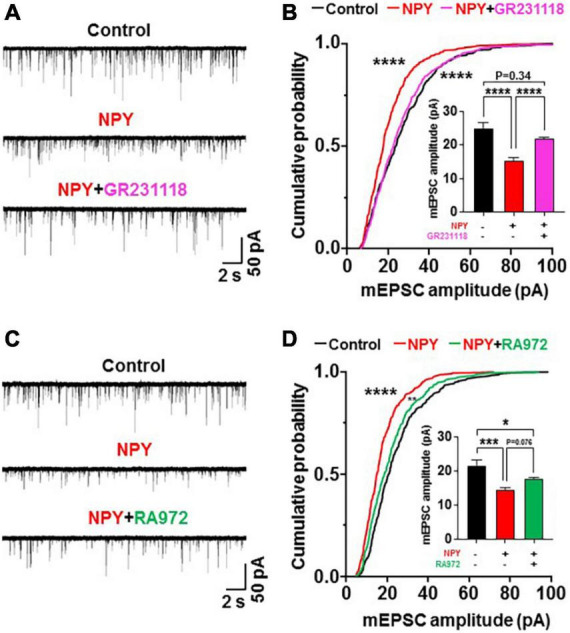
Neuropeptide Y (NPY) depresses miniature excitatory postsynaptic current (mEPSC) by interacting with Y1 receptor (Y1R) but not Y5R. Hypothalamic slices from OXT mRFP rats were pretreated for 3 h with NPY in the absence and presence of the Y1R or Y5R antagonist. **(A)** Incubation with NPY depressed mEPSC, and this effect was blocked by simultaneous treatment with the Y1R antagonist GR231118 (0.5 μM). **(B)** Incubation with NPY left-shifted the cumulative probability distribution and reduced the average of mEPSC amplitudes (insert). These effects were blocked by simultaneous treatment with GR231118. *n* = 8–9. ****P < 0.0001 between control vs. NPY and NPY vs. NPY + GR231118 by Kruskal-Wallis test followed by *post-hoc* Dunn’s test. In insert ****P < 0.0001, and *P* = 0.34 between control vs. NPY + GR231118 by one-way ANOVA followed by *post-hoc* Tukey’s test. **(C)** NPY without and with Y5R antagonist RA972 (0.1 μM) depressed mEPSC in paraventricular nucleus oxytocin neurons. **(D)** NPY without and with RA972 significantly left-shifted the cumulative probability distribution and reduced the average of mEPSC amplitudes (insert) in PVN OXT neurons. *n* = 8–9. ****P < 0.0001 between control vs. NPY, and **P < 0.01 between control vs. NPY + RA972 by Kruskal-Wallis test followed by *post-hoc* Dunn’s test. In insert *P < 0.05, ***P < 0.001, and *P* = 0.076 between NPY vs. NPY + RA972 by one-way ANOVA followed by *post-hoc* Tukey’s test.

Y1 receptor and Y5R in the central nervous system have been implicated in the promotion of feeding by NPY (20). The role of Y1R was assessed by examining the effect of the Y1R antagonist, GR231118 (0.5 μM), on the action of NPY. Treatment with NPY decreased the amplitude of mEPSC, and this effect was blunted by simultaneous treatment with GR231118 (Figure 4A) in OXT neurons. GR231118 also significantly counteracted the NPY action to left-shift the mEPSC distribution (*P* < 0.0001 in Control vs. NPY, *P* < 0.0001 in NPY vs. NPY + GR231118, *P* > 0.99 in Control vs. NPY + GR231118, Kruskal-Wallis test followed by Dunn’s test) and decrease the average of mEPSC amplitude (Control vs. NPY, 24.87 ± 1.68 vs. 15.49 ± 0.71 pA, *P* < 0.0001; NPY vs. NPY and GR231118, 15.49 ± 0.71 vs. 22.04 ± 0.38 pA, *P* < 0.0001; Control vs. NPY + GR231118, *P* = 0.34, one-way ANOVA followed by *post-hoc* Tukey’s test, *n* = 8–9; Figure 4B). Neither NPY nor GR231118 significantly altered the mEPSC frequency (Supplementary Figure 2C). In contrast, treatment with the Y5R antagonist RA972 (0.1 μM) did not significantly affect the actions of NPY on mEPSC, its amplitude distribution and average amplitude (*P* = 0.076; Figures 4C,D). Therefore, Y1R, but not Y5R, is implicated in the action of NPY on mEPSC in OXT neurons. Without administration of NPY, neither GR231118 nor RA972 affected the mEPSC amplitude and frequency in OXT neurons (Supplementary Figure 1). Thus, the elevated NPY due to 24 h FR or exogenous administration interacts with Y1R to depress mEPSC by inhibiting NMDAR in PVN OXT neurons.

### Twenty four hour food restriction *via* Y1 receptor depresses miniature excitatory postsynaptic current in paraventricular nucleus oxytocin neurons and induces hyperphagia

**FIGURE 5:**
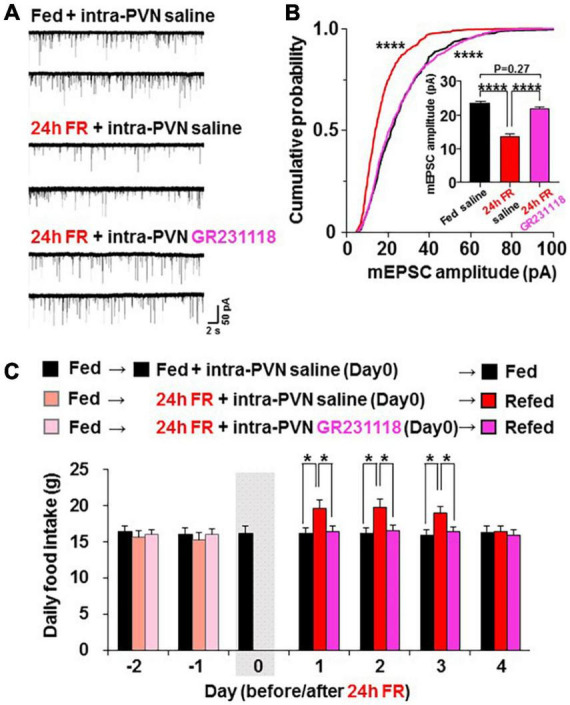
Twenty four hours food restriction (24 h FR) depresses miniature excitatory postsynaptic current (mEPSC) and induces hyperphagia *via* Y1 receptor (Y1R). **(A)** 24 h FR reduced mEPSC in paraventricular nucleus (PVN) oxytocin (OXT) neurons, and this effect was counteracted by the intra- paraventricular nucleus (PVN) injection of the Y1R antagonist GR231118 (10 μg/5 μl, 0.3 μl) during the 24 h FR period. **(B)** Twenty four hours FR left-shifted the cumulative probability distribution and reduced the average of mEPSC amplitudes (insert), and these effects were blocked by simultaneous treatment with GR231118. *n* = 9–11. ****P < 0.0001 between fed vs. 24 h FR and between 24 h FR vs. 24 h FR + GR231118 groups by Kruskal-Wallis test followed by *post-hoc* Dunn’s test. ****P < 0.0001 and *P* = 0.27 between fed vs. fasted + GR231118 by one-way ANOVA followed by *post-hoc* Tukey’s test. **(C)** Daily food intake was significantly elevated on Days 1–3 in oxytocin mRFP rats treated with 24 h FR and intra-PVN injected with saline compared with fed rats intra-PVN injected with saline, and this elevation was completely blocked in rats treated with 24 h FR and intra-PVN injected with GR231118. The gray shadow indicates the day of 24 h FR. *n* = 6. *P < 0.05 by two-way ANOVA followed by *post-hoc* Sidak’s test.

We examined whether the NPY-Y1 system is also involved in the effects of 24 h FR to regulate synaptic plasticity and feeding behavior. Two weeks after surgery for guide cannulation into the PVN, OXT-mRFP rats were fasted from 10:00 on Day 0 to 10:00 on Day 1 followed by re-feeding from Day 1 (Figure 1A). On Day 0, intra-PVN injection of saline (0.9%) or Y1R antagonist GR231118 (10 μg/5 μl, 0.3 μl) was performed at 19:00, 30 min before the onset of the dark cycle (Figure 1A). Hypothalamic slices were prepared from fed rats receiving saline, 24 h FR rats receiving saline, and 24 h FR rats receiving GR231118. Then, mEPSC in PVN OXT neurons was measured at 11:00–13:00 on Day 1 (Figure 1A). After 24 h FR, compared to the fed control, the mEPSC amplitude was decreased (Figure 5A) with its distribution left-shifted (*P* < 0.0001 in Fed vs. 24 FR, *P* < 0.0001 in 24 FR vs. 24 FR + GR231118, *P* > 0.99 in Fed vs. 24 FR + GR231118, Kruskal-Wallis test followed by Dunn’s test) and average reduced (Fed vs. 24 h FR, 23.37 ± 0.65 vs. 13.81 ± 0.70 pA, *P* < 0.0001; 24 h FR vs. 24 h FR + GR231118, 13.81 ± 0.70 vs. 21.94 ± 0.56 pA, *P* < 0.0001; Fed vs. 24 h FR + GR231118, *P* = 0.27, one-way ANOVA followed by *post-hoc* Tukey’s test, *n* = 9–11; Figure 5B). These changes were completely counteracted by intra-PVN injection of the Y1 antagonist GR231118 (Figures 5A,B). Neither 24 h FR nor GR231118 significantly altered the mEPSC frequency (Supplementary Figure 2E). Daily food intake was measured at 10:00 in the period from 2 days before 24 h FR (Day-2) through Day 4 after re-feeding (Figure 5C). Significant elevation of daily food intake was observed on Day 1 to Day 3 in 24 h FR rats compared with fed rats, and this elevation was completely blocked in 24 h FR rats injected with GR231118 during 24 h FR on Day 1–3 (Fed vs. 24 h FR vs. 24 h FR + GB231118; Day 1, 16.10 ± 0.76 vs. 19.61 ± 0.97 vs. 16.43 ± 0.74 g; Day 2, 16.21 ± 0.71 vs. 19.73 ± 0.99 vs. 16.49 ± 0.75 g; Day3, 15.93 ± 0.74 vs. 18.995 ± 0.92 vs. 16.38 ± 0.71 g, *P* < 0.05 in Fed vs. 24 h FR and 24 h FR vs. 24 h FR + GR231118, two way-ANOVA followed by Sidak’s test, *n* = 6; Figure 5C). These data implicate the NPY-Y1R system in the fasting-evoked stimulation of feeding and synaptic alteration, consistent with a previous report that NPY ablation impairs the re-feeding response to fasting (21).

### 50% food restriction induces miniature excitatory postsynaptic current depression, early hyperphagia and sustained weight control

**FIGURE 6:**
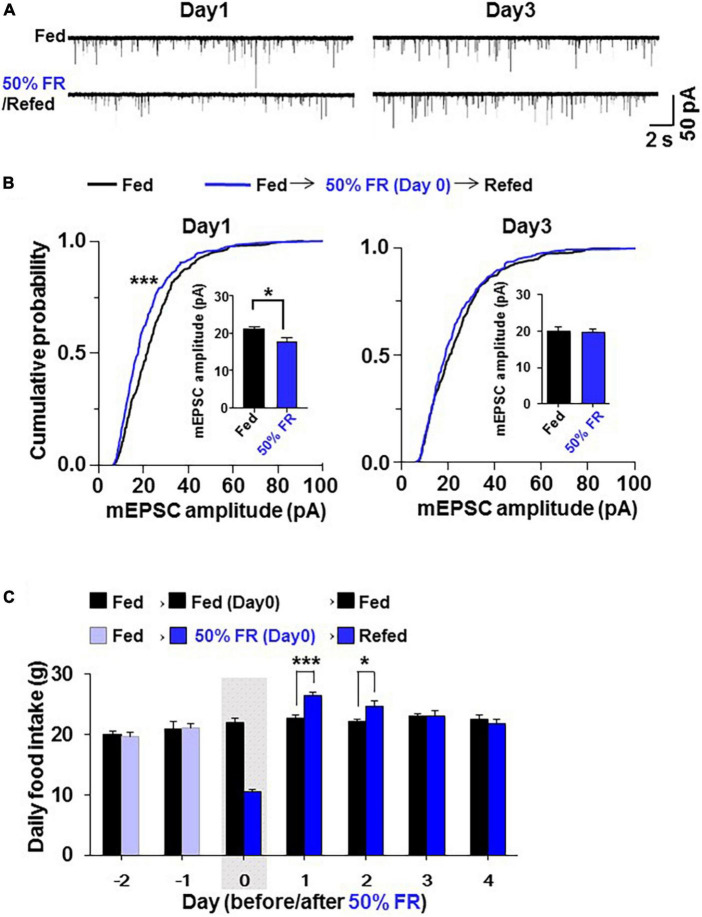
50% food restriction (FR) induces synaptic depression and hyperphagia less potently than 24 h FR. In the 50% FR group, 50% of the food consumed under the control ad-lib fed condition was given on Day 0 to oxytocin (OXT) mRFP rats, followed by feeding and patch-clamp studies on Days 1–4. **(A)** The effect of 50% FR, compared to the fed condition, on miniature excitatory postsynaptic current (mEPSC) in paraventricular nucleus (PVN) OXT neurons. **(B)** 50% FR left-shifted cumulative probability distribution and reduced the average of mEPSC amplitudes (insert) in PVN OXT neurons on Day 1 but not Day 3 of re-feeding. *n* = 7. ***P < 0.001 by Kolmogorov–Smirnov test. *P < 0.05 by unpaired *t*-test. **(C)** 50% FR, compared to the fed condition, resulted in elevated daily food intake on Days 1 and 2 but not Days 3 and 4 of re-feeding. The gray shadow indicates the day of 50% FR. *n* = 5–6. *P < 0.05 and ***P < 0.001 by two-way ANOVA followed by *post-hoc* Sidak’s test.

**FIGURE 7:**
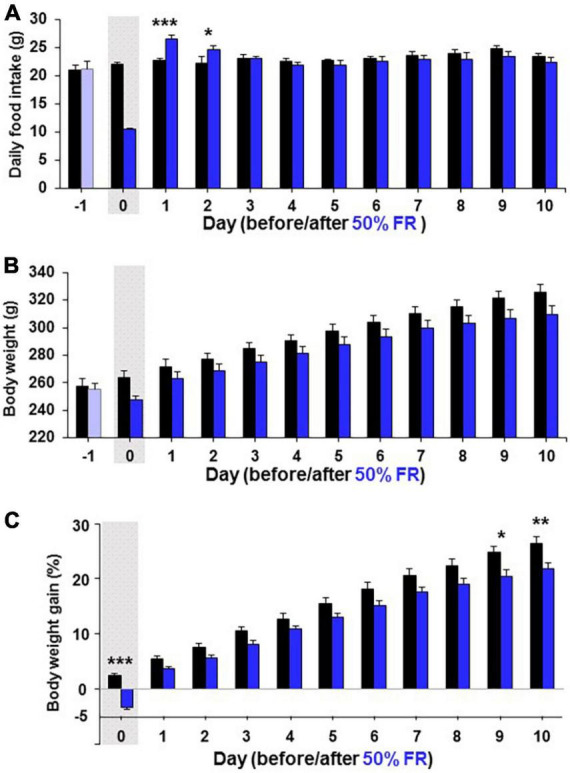
50% FR induces continuous weight reduction without rebound. Long-term effects of 50% FR on daily food intake **(A)**, body weight **(B)**, and body weight gain **(C)** in Wistar rats. The body weight gain was determined by the change from the weight on Day-1. The gray shadows indicate the day of 50% FR. *n* = 5–6. *P < 0.05, **P < 0.01, and ***P < 0.001 between 50% FR vs. fed groups by two-way ANOVA followed by *post-hoc* Sidak’s test.

Since 24 h FR resulted in late hyperphagia and weight rebound, a different fasting condition that does not induce late hyperphagia and weight rebound was desirable in dieting. Therefore, a second milder fasting regimen, 50% FR, was examined. On Day 1 after 50% FR, mEPSC amplitude in PVN OXT neurons was inhibited (Figure 6A) with its distribution significantly (*P* < 0.0001, Kolmogorov–Smirnov test) left-shifted and its average significantly reduced (Fed vs. 50% FR; Day 1, 21.03 ± 0.80 vs. 17.77 ± 1.12 pA, *P* < 0.05; Day 3, 20.16 ± 0.99 vs. 19.84 ± 0.82 pA, *P* = 0.89, by unpaired *t*-test, *n* = 7; Figure 6B). The mEPSC inhibition was not observed on Day 3 (Figures 6A,B). 50% FR did not significantly alter the mEPSC frequency on Days 1 and 3 (Supplementary Figure 2F). In parallel, 50% FR significantly increased daily food intake on Days 1–2 (Fed vs. 50% FR; Day 1, 22.69 ± 0.51 vs. 26.53 ± 0.54 g, *P* < 0.001; Day 2, 22.208 ± 0.51 vs. 24.65 ± 0.87 g, *P* < 0.05, two way-ANOVA followed by Sidak’s test, *n* = 5–6) but not on Day 3 (Figure 6C). Thus, the 50% FR-induced mEPSC inhibition and hyperphagia took place on Day 1 and disappeared on Day 3, showing a similar time course, which was shorter lasting than that of 24 h FR (Figures 6A–C vs. Figures 1B–D). After 50% FR, daily food intake was significantly increased on Days 1–2, followed by no changes from thereon (Figure 7A). Weight tended to be lower on Days 0–10 (Figure 7B). Notably, the weight gain tended to be lower on Days 1–8 and reached significant reduction on Days 9–10 (Fed vs. 50% FR; Day9, 24.75 ± 1.16 vs. 20.52 ± 1.07%, *P* < 0.05; Day 10, 26.40 ± 1.17 vs. 21.86 ± 1.08%, *P* < 0.01, two way-ANOVA followed by Sidak’s test, *n* = 5–6; Figure 7C).

### Hysteresis in the early “synaptic depression—hyperphagia” relationship underlies late outcomes

**FIGURE 8:**
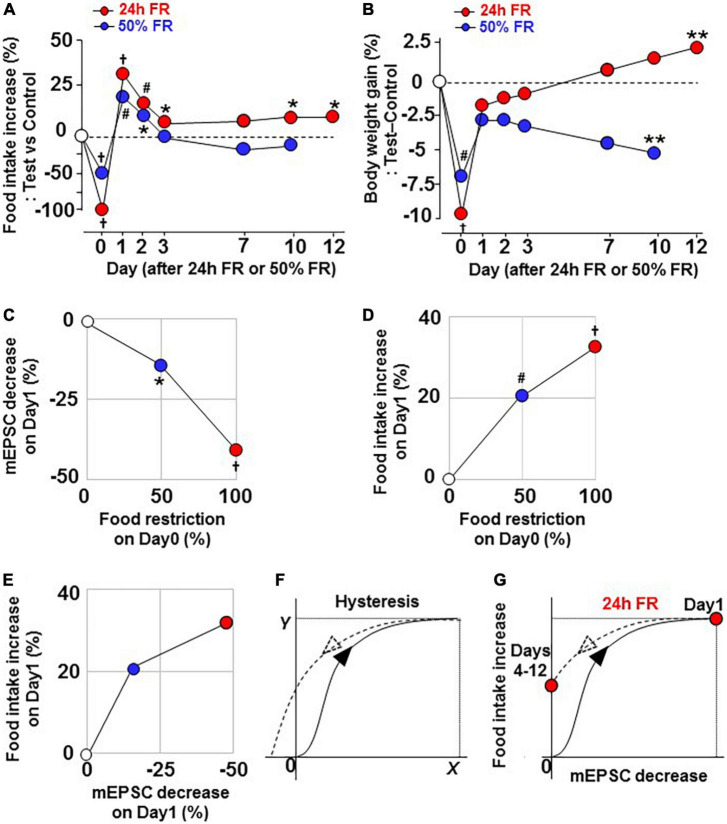
Twenty four hours FR, but not 50% FR, induces rebound through hysteresis in the ‘miniature excitatory postsynaptic current inhibition—hyperphasia’ relationship. Data are re-plotted from Figures 2A,C, 7A,C. **(A,B)** Comparable effects of 24 h FR and 50% FR on changes in daily food intake **(A)** and body weight gain **(B)**. Twenty four hours FR, but not 50% FR, induced increases in daily food intake and body weight gain on Day 10 and later. **(C**,**D)** Relationship of food restriction (%) on Day 0 to mEPSC decrease (%) on Day 1 **(C)** and to food intake increase (%) on Day 1 **(D)**. **(E)** Relationship between mEPSC decrease (%) on Day 1 and food intake increase (%) on Day 1. **(F)** Hysteresis, in the “*X–Y”* relationship. **(G)** Hysteresis, presumed in the “mEPSC decrease–food intake increase” relationship and consequent upward shift of food intake on Days 4–12 after 24 h FR. *P < 0.05, **P < 0.01, ^#^*P* < 0.001, and **^†^***P* < 0.0001 between 24 h FR or 50% FR vs. fed groups by two-way ANOVA followed by *post-hoc* Sidak’s test.

Twenty four hour FR induced hyperphagia on Days 1–3 and again on Days 9–12 associated with increases in the weight gain on Days 12–17 (Figures 2A–C). In contrast, 50% FR induced hyperphagia on Days 1–2 associated with reductions in the weight gain on Days 9–10 (Figures 7A–C). Thus, the extent of FR that elicits the initial response of the feeding regulatory system influences the late outcome. Hence, we compared the early vs. late changes in food intake and weight under the 24 h FR and 50% FR regimens. As shown in Figures 8A,B, in the 24 h FR group, stronger reductions in food intake and weight on Day 0 were followed by greater mEPSC depression on Day 1 (Figure 5B vs. Figure 6B) and overeating on Days 1–3, and these early changes appeared to be linked to the late hyperphagia and rebound weight gain on Days 10–12. Furthermore, a transverse data analysis on Day 1 after 24 h FR and 50% FR, showed that 24 h FR compared to 50% FR induced a 2.8-fold greater decrease in mEPSC on OXT neurons (Figure 8C) and a 1.6-fold greater increase in food intake on Day 1 (Figure 8D). Consequently, the relationship between decreased mEPSC and increased food intake showed a saturation curve (Figure 8E), the kinetics of which can induce hysteresis (22, 23) (Figure 8F). This hysteresis may serve as a mechanism to upwardly shift the setpoint for regulation of feeding after 24 h FR (Figure 8G) and involve the alteration of glutamatergic synapses (Figures 3C–F). This is consistent with previous reports of hysteresis for the NMDAR channel regulation in mouse forebrain neurons (24) and for the fasting-induced plasticity in glutamatergic synapses on mouse ARC NPY/AgRP neurons (22, 23).

## Discussion

**FIGURE 9:**
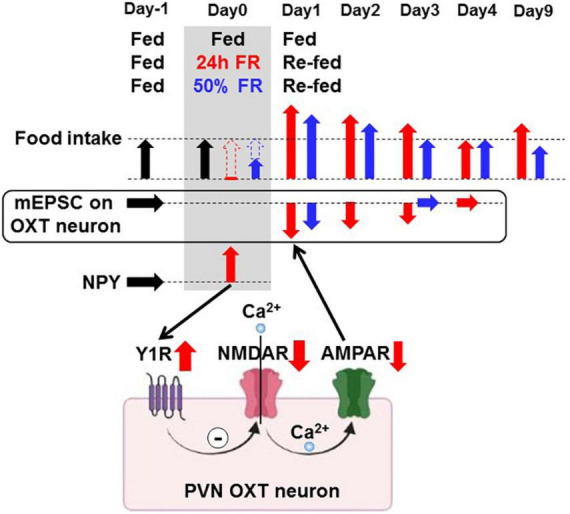
Proposed mechanisms of food restriction-induced synaptic depression in paraventricular nucleus oxytocin neurons accompanied by hyperphagia. Neuropeptide Y, either elevated by 24 h/50% FR or administered, induces the Y1 receptor-mediated inhibition of glutamatergic synaptic input on PVN OXT neurons, the pathway possibly involving NMDAR and AMPAR. 24 h FR and 50% FR evoke this neuronal signaling and induce acute hyperphagia for 2–3 days on re-feeding. Twenty four hour FR but not 50% FR also induces late hyperphagia on Day 9 and later.

In this study, NPY depressed mEPSC on PVN OXT neurons by interacting with Y1R and attenuating glutamatergic synapses (Figure 9). Moreover, 24 h FR depressed mEPSC on OXT neurons and increased daily food intake: these effects took place for 3 days after re-feeding and were completely blocked by the Y1R antagonist focally injected into PVN during 24 h FR. These results demonstrate that the 24 h FR-driven Y1R-mediated mEPSC inhibition on anorexigenic OXT neurons in PVN likely serves to induce early-phase hyperphagia (Figure 9).

This study shows the fasting-induced depression of excitatory synaptic transmission on PVN OXT neurons *via* NPY-Y1R, a mechanism that induces rapid hyperphagia upon re-feeding and serves for restoration of energy homeostasis. The pathways upstream and downstream of this NPY-OXT neuronal signaling remain to be elucidated. However, the upstream pathways possibly involve ghrelin, a hunger hormone that stimulates appetite and contributes to hyperphagia and rebound weight gain (25, 26). The downstream pathways could involve the projection of PVN OXT neurons to the hindbrain including the nucleus tractus solitarius (5) and/or to ARC POMC neurons (27).

Another notable finding is that although the 24 h FR and 50% FR exerted common short-term effects of acute hyperphagia and weight recovery by Day 3 of re-feeding, they resulted in different long-term outcomes. On Day 9 and later, food intake and weight gain were increased in the 24 h FR groups, but continuously reduced in the 50% FR groups. This study strongly suggests that the deep depression of synaptic input on OXT neurons is a factor that couples 24 h FR to the late hyperphagia and weight gain (Figure 9). The plasticity of the OXT system and its (patho)physiological significance were also previously reported in a study showing the parallel rescue of the OXT response and social behavior in a mouse model of autism (28). It should also be noted that the rebound weight gain would additionally involve reduced energy expenditure. In support of this, re-feeding after fasting increases food efficiency (29) and activates the melanocortin signaling that inhibits TRH neurons and thereby heat production (21, 30).

## Conclusion

In conclusion, strong rewiring of the synaptic plasticity on OXT neurons by 24 h FR produces hysteresis (Figures 8E,F), which upwardly shifts the setpoint for the regulation of feeding (Figure 8G) and consequently that for weight regulation, leading to long-lasting hyperphagia and weight rebound (Figures 8A,B). In contrast, moderate conditions of FR, illustrated by 50% FR, that optimally alter the synaptic plasticity without hysteresis, achieve sustainable control of weight without rebound. These opposite outcomes between 24 h FR vs. 50% FR imply failure vs. success in the context of dieting (Figure 9). These results highlight the NPY-Y1R-driven glutamatergic synaptic input on OXT neurons as key players for the post-fasting restoration of energy homeostasis and long-term outcome of weight control, and as a potential target for preventing and/or treating rebound after dieting.

## Data availability statement

The original contributions presented in the study are included in the article/Supplementary material, further inquiries can be directed to the corresponding author.

## Ethics statement

Animal experiments were carried out under approval by the Jichi Medical University Animal Care and Use Committee and by the Committee on Animal Experimentation of Kobe University.

## Author contributions

SS, LW, GS, and TY designed the study and wrote the manuscript. LW, SS, and SL conducted the experiments. YU provided OXT-mRFP transgenic and AVP-GFP transgenic rats. YS participated in discussion. TY supervised the work. All authors contributed to the article and approved the submitted version.

## Supplementary material

Supplementary Figure 1 Without NPY administration, neither the Y1R antagonist nor the Y5R antagonist affected mEPSC amplitude and frequency in PVN OXT neurons. Hypothalamic slices were pretreated for 3 h without or with the Y1R antagonist GR231118 (0.5 μM) or the Y5R antagonist RA972 (0.1 μM). **(A)** mEPSC in PVN OXT neurons. Upper and lower traces are from two distinct OXT neurons. **(B–D)** Cumulative probability distribution **(B)**, average amplitude **(C),** and frequency **(D)** of mEPSC in PVN OXT neurons. Neither GR231118 nor RA972 altered mEPSC amplitude: *P* = 0.33 between control vs. GR231118, *P* = 0.30 between control vs. RA972, and *P* = 0.68 between GR231118 vs. RA972 by one-way ANOVA followed by *post-hoc* Tukey’s test, *n* = 9 (**c**). Neither GR231118 nor RA972 altered mEPSC frequency: *P* = 0.65 between control vs. GR231118, *P* = 0.86 between control vs. RA972, and *P* = 0.66 between GR231118 vs. RA972 by one-way ANOVA followed by *post-hoc* Tukey’s test, *n* = 9 **(D)**.

Supplementary Figure 2 Effects of various treatments on mEPSC frequency in PVN OXT neurons. **(A)** 24 h FR did not significantly alter mEPSC frequency in PVN OXT neurons on Days 1, 3, and 4. *P* = 0.96 (Day 1), *P* = 0.83 (Day 3), and *P* = 0.84 (Day 4) between Fed vs. 24 h FR by unpaired *t*-test, *n* = 6–9. **(B)** Neither NPY nor AP5 significantly altered mEPSC frequency in PVN OXT neurons. *P* = 0.42 between control vs. NPY and *P* = 0.85 between control vs. AP-5 by unpaired *t*-test, *n* = 6–8. **(C)** NPY and NPY + GR231118 did not significantly alter mEPSC frequency in PVN OXT neurons. *P* = 0.20 between control vs. NPY, *P* = 0.72 between control vs. NPY + GR231118 and *P* = 0.61 between NPY vs. NPY + GR231118 by one-way ANOVA followed by *post-hoc* Tukey’s test, *n* = 8–9. **(D)** NPY and NPY + RA972 did not significantly alter mEPSC frequency in PVN OXT neurons. *P* = 0.88 between control vs. NPY, *P* = 0.73 between control vs. NPY + RA972 and *P* = 0.94 between NPY vs. NPY + RA972 by one-way ANOVA followed by *post-hoc* Tukey’s test, *n* = 8–9. **(E)** 24 h FR and 24 h FR + GR231118 did not significantly alter mEPSC frequency in OXT neurons. *P* = 0.34 between Fed vs. 24 h FR, *P* = 0.41 between 24 h FR vs. 24 h FR + GR231118, and *P* = 0.78 between Fed vs. 24 h FR + GR231118 by one-way ANOVA followed by *post-hoc* Tukey’s test, *n* = 9–11. **(F)** 50% FR did not significantly alter mEPSC frequency in PVN OXT neurons on Days 1 and 3. *P* = 0.81 (Day 1) and *P* = 0.89 (Day 3) between Fed vs. 50% FR by unpaired *t*-test, *n* = 7. These mEPSC frequency graphs are paired with corresponding mEPSC amplitude graphs presented in the Figures specified on top of graphs.
