# Supplementary figures and images for: Fasting inhibits excitatory synaptic input on paraventricular oxytocin neurons via neuropeptide Y and Y1 receptor, inducing rebound hyperphagia, and weight gain
Source: Front Nutr. 2022 Oct 19;9:994827. doi: 10.3389/fnut.2022.994827 (PMC9627337; doi:10.3389/fnut.2022.994827)

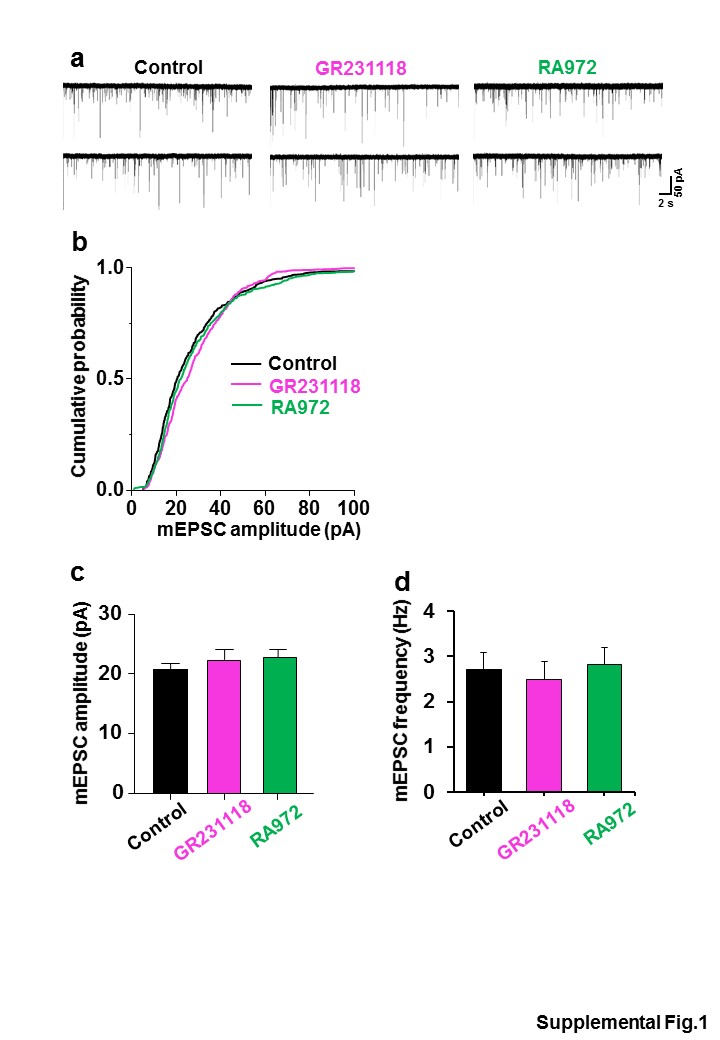

Supplement: Supplementary Figure 1 — Without NPY administration, neither the Y1R antagonist nor the Y5R antagonist affected mEPSC amplitude and frequency in PVN OXT neurons. Hypothalamic slices were pretreated for 3 h without or with the Y1R antagonist GR231118 (0.5 μM) or the Y5R antagonist RA972 (0.1 μM). (A) mEPSC in PVN OXT neurons. Upper and lower traces are from two distinct OXT neurons. (B–D) Cumulative probability distribution (B), average amplitude (C), and frequency (D) of mEPSC in PVN OXT neurons. Neither GR231118 nor RA972 altered mEPSC amplitude: P = 0.33 between control vs. GR231118, P = 0.30 between control vs. RA972, and P = 0.68 between GR231118 vs. RA972 by one-way ANOVA followed by post-hoc Tukey’s test, n = 9 (c). Neither GR231118 nor RA972 altered mEPSC frequency: P = 0.65 between control vs. GR231118, P = 0.86 between control vs. RA972, and P = 0.66 between GR231118 vs. RA972 by one-way ANOVA followed by post-hoc Tukey’s test, n = 9 (D). [file Image_1.jpg]

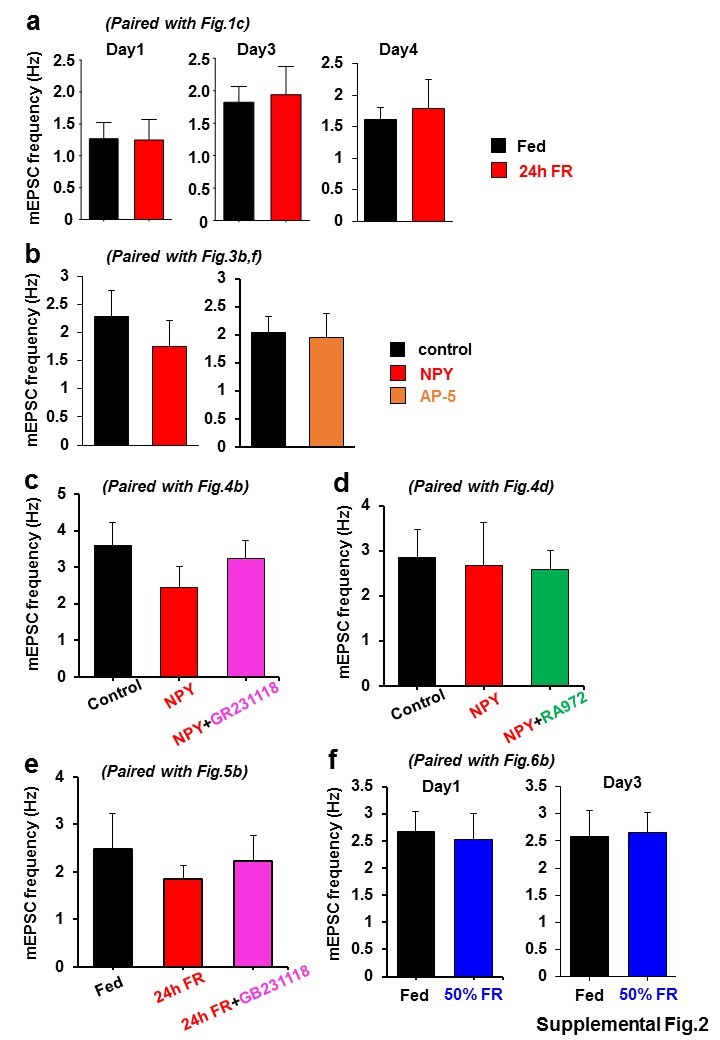

Supplement: Supplementary Figure 2 — Effects of various treatments on mEPSC frequency in PVN OXT neurons. (A) 24 h FR did not significantly alter mEPSC frequency in PVN OXT neurons on Days 1, 3, and 4. P = 0.96 (Day 1), P = 0.83 (Day 3), and P = 0.84 (Day 4) between Fed vs. 24 h FR by unpaired t-test, n = 6–9. (B) Neither NPY nor AP5 significantly altered mEPSC frequency in PVN OXT neurons. P = 0.42 between control vs. NPY and P = 0.85 between control vs. AP-5 by unpaired t-test, n = 6–8. (C) NPY and NPY + GR231118 did not significantly alter mEPSC frequency in PVN OXT neurons. P = 0.20 between control vs. NPY, P = 0.72 between control vs. NPY + GR231118 and P = 0.61 between NPY vs. NPY + GR231118 by one-way ANOVA followed by post-hoc Tukey’s test, n = 8–9. (D) NPY and NPY + RA972 did not significantly alter mEPSC frequency in PVN OXT neurons. P = 0.88 between control vs. NPY, P = 0.73 between control vs. NPY + RA972 and P = 0.94 between NPY vs. NPY + RA972 by one-way ANOVA followed by post-hoc Tukey’s test, n = 8–9. (E) 24 h FR and 24 h FR + GR231118 did not significantly alter mEPSC frequency in OXT neurons. P = 0.34 between Fed vs. 24 h FR, P = 0.41 between 24 h FR vs. 24 h FR + GR231118, and P = 0.78 between Fed vs. 24 h FR + GR231118 by one-way ANOVA followed by post-hoc Tukey’s test, n = 9–11. (F) 50% FR did not significantly alter mEPSC frequency in PVN OXT neurons on Days 1 and 3. P = 0.81 (Day 1) and P = 0.89 (Day 3) between Fed vs. 50% FR by unpaired t-test, n = 7. These mEPSC frequency graphs are paired with corresponding mEPSC amplitude graphs presented in the Figures specified on top of graphs. [file Image_2.jpg]
